# A Hardware System for Synchronous Processing of Multiple Marine Dynamics MEMS Sensors

**DOI:** 10.3390/mi13122135

**Published:** 2022-12-02

**Authors:** Junmin Jing, Zengxing Zhang, Zhiwei Liao, Bin Yao, Yuzhen Guo, Wenjun Zhang, Yanbo Xu, Chenyang Xue

**Affiliations:** 1 Key Laboratory of Instrumentation Science and Dynamic Measurement, Ministry of Education, North University of China, Taiyuan 030051, China; 2 School of Aerospace Engineering, Xiamen University, Xiamen 361005, China; 3 Tan Kah Kee Innovation Laboratory, Xiamen 361005, China

**Keywords:** FAT file system, platinum film resistance, four-electrode platinum thin-film array, piezoresistive pressure sensor, turbulent solver

## Abstract

Temperature, depth, conductivity, and turbulence are fundamental parameters of marine dynamics in the field of ocean science. These closely correlated parameters require time-synchronized observations to provide feedback on marine environmental problems, which requires using sensors with synchronized power supply, multi-path data solving, recording, and storage performances. To address this challenge, this work proposes a hardware system capable of synchronously processing temperature, depth, conductivity, and turbulence data on marine dynamics collected by sensors. The proposed system uses constant voltage sources to excite temperature and turbulence sensors, a constant current source to drive a depth sensor, and an alternating current (AC) constant voltage source to drive a conductivity sensor. In addition, the proposed system uses a high-precision analog-digital converter to acquire the direct current (DC) signals from temperature, depth, and turbulence sensors, as well as the AC signals from conductivity sensors. Since the sampling frequency of turbulence sensors is different from that of the other sensors, the proposed system stores the generated data at different storage rates as multiple-files. Further, the proposed hardware system manages these files through a file system (file allocation tab) to reduce the data parsing difficulty. The proposed sensing and hardware logic system is verified and compared with the standard conductivity-temperature-depth measurement system in the National Center of Ocean Standards and Metrology. The results indicate that the proposed system achieved National Verification Level II Standard. In addition, the proposed system has a temperature indication error smaller than 0.02 °C, a conductivity error less than 0.073 mS/cm, and a pressure error lower than 0.8‰ FS. The turbulence sensor shows good response and consistency. Therefore, for observation methods based on a single point, single line, and single profile, it is necessary to study multi-parameter data synchronous acquisition and processing in the time and spatial domains to collect fundamental physical quantities of temperature, salt, depth, and turbulence. The four basic physical parameters collected by the proposed system are beneficial to the in-depth research on physical ocean motion, heat transfer, energy transfer, mass transfer, and heat-energy-mass coupling and can help to realize accurate simulation, inversion, and prediction of ocean phenomena.

## 1. Introduction

Marine turbulence is a type of motion that corresponds to three-dimensional irregular laminar shear and fluid vortex. Among the turbulence types, medium- and small-scale turbulences affect the seawater transfer of momentum and heat [1]. Turbulence is an essential driver for ocean mixing [2,3], temperature regulation, and salinity distribution in blocks, forming a water layer density gradient and changing the lateral diffusion of density. In addition, the uneven distribution of temperature and salinity in the three-dimensional block of seawater also affects the formation, development, and annihilation of turbulence [4]. The temperature, salinity, and water depth are inherently coupled and related as three crucial parameters of marine dynamics. For instance, seawater salinity is calculated based on depth, conductivity, and temperature data [5]. Therefore, for a thorough comprehension of turbulent mixing, energy flow dissipation [6], transition layer evolution, and prediction of ocean current changes, it is essential to acquire data on seawater temperature, electrical conductivity, depth, and turbulence in a spatiotemporal synchronous manner.

Ocean dynamics observations mainly rely on ocean profilers, such as thermometers, turbulence meters, and current meters. The temperature and salt depth meters (CTDs) can be divided into cable-integrated and data-self-contained CTDs; the former are suitable for navigation and mobile observations, whereas the latter are suitable for distributed, disposable, and long-term observations [7]. For turbulometers, multiple piezoelectric strain-gauge probes are required to meet the vector observation requirements. In contrast, the CTD integrated with the turbulence measurement function has a large volume and high energy consumption and cost, which makes it challenging to meet the requirements of rapid and large-area observations, such as distributed and disposable monitoring. Therefore, it is necessary to develop a self-contained, small-sized, low-cost, 4-parameter spatiotemporal synchronous detection system that can be rapidly deployed.

To address the aforementioned needs, this work develops a miniaturized, self-contained, four-parameter simultaneous acquisition and storage system that integrates temperature, depth, salinity, and turbulence measurement functions. The proposed system has a size of only 9 cm × 2.5 cm, which is easy to pack. The proposed system includes several measurement modules, each of which can be independently activated, and different sensing heads can be selected according to specific application environments. Moreover, each sensor module is calibrated. When the temperature module is in the range of −5–40 °C, the indication error is within 0.02 °C; when the pressure module is in the range of 0–3 MPa, the appeared indication error is only 2.4 kPa, and the full-scale error is only 0.8‰. In the range from zero to 70 mS/cm, the conductivity measurement error is less than 0.073 mS/cm; at the scale of flow velocity less than 1 m/s, the *x* and *y* axes of the vector turbulence sensor are highly consistent, which indicate high directivity degree. The proposed hardware system can help to further understanding and research of turbulent mixing, energy flow dissipation, transition layer evolution, and prediction of ocean current changes.

## 2. Result and Discussion

### 2.1. Design

**Figure 1:**
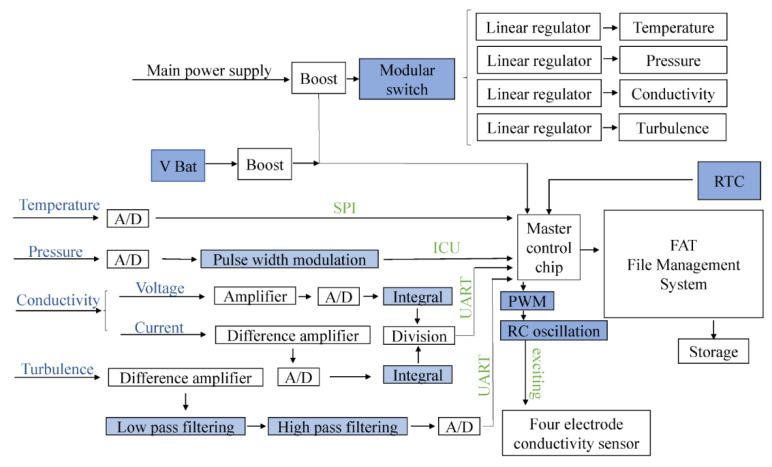
The overall hardware logic system.

The integrated structure of the design is shown in Figure 1, where it can be seen that the overall framework can be divided into three main sections: power module, sensor parameter acquisition module, and data storage module.

The power module has two sub-modules, the main power working module and the backup power clock module. After the system runs by turning on the main switch, the storage module begins to function as normal. The system selects the required module functions using a modular switch to match the corresponding sensor. Conversely, once the main power switch is turned off, the V Bat backup provides power to the central control chip, which only works with the real-time clock (RTC), to ensure the stored-file time matches the actual time.

The sensor parameter acquisition module can be further divided into temperature, pressure, conductivity, and turbulence sensor modules. According to actual usage requirements, the sensor parameter acquisition module selects a relevant sensing function and uses a platinum-resistance-based device for temperature measurement, a four-electrode conductivity sensor for conductivity measurements, piezoresistive pressure for sensitive pressure measurements, and a vector turbulence sensor for turbulence measurements.

Particularly, platinum thermal resistance is used as the temperature-sensitive probe in the temperature module. Among various available platinum thermal resistance types (e.g., PT100, PT1000, and PT5000), a PT1000, which has a resistance of 1 kOhm at the zero Celsius temperature, is adopted in this work. The PT1000 resistance is sensitive to temperature within a specific temperature range, where the PT1000 has features of high linearity, short response time, and high repeatability. In addition, the pressure module has a piezoresistive pressure sensor with high precision, high-frequency response, low hysteresis, and high reliability. Similarly, the conductivity module adopts a four-electrode conductivity sensor, which has high sensitivity, strong anti-pollution ability, and can eliminate the influence of electrode polarization. Finally, the turbulence module uses a self-developed MEMS vector turbulence sensor, which has a high sensitivity.

The data storage module obtains files through the File Allocation Tab (FAT) file management system. The advantage of the FAT file system is that it does not depend on the hardware platform. The scanning frequency of the temperature, pressure, and conductivity sensors is 1 Hz, while that of the turbulence sensor is higher than 1 kHz. Therefore, this design uses the FAT file management system to store the data acquired by the turbulence and other sensors in multiple files. It should be noted that turbulence sensor data are stored in one folder, while other parameters are stored in another folder. In addition, for low-frequency measurements, this design packs and stores data in seconds. On the contrary, for high-frequency turbulence parameters, this design stores data in milliseconds. To reduce data parsing difficulty and enhance data readability in the process of data packaging and storing, the proposed design places minutes and seconds after the frame header, takes hours as a secondary directory of a file, and uses data as the primary directory of the file.

### 2.2. Hardware

#### 2.2.1. Temperature Sensor Conditioning Circuit

The temperature conditioning module uses a ceramic platinum thermal thin film resistor as a sensitive unit of a temperature sensor [8,9,10,11].

**Figure 2:**
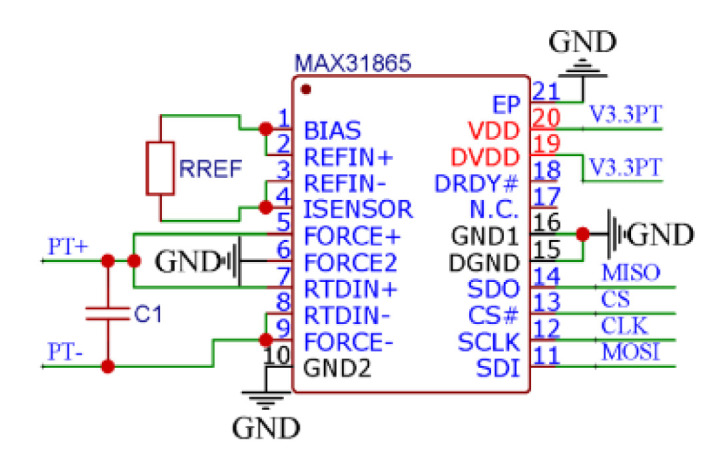
Schematic diagram of the temperature conditioning circuit.

The temperature module converts the resistance changes into voltage changes for the purpose of easy signal processing to measure the platinum Resistance Temperature Detector (RTD). Therefore, it is necessary to ensure that the platinum RTD current is consistent with the reference resistance current. Thus, a MAX31865 conversion chip from MAXIM, which meets the requirements of the temperature module for efficient conversion of platinum thermal resistance, is used in this study. In addition, the MAX31865 conditioning chip has low system power consumption and 15-bit analog-digital converter (ADC) resolution, which enables it to maintain high precision under the premise of low power consumption when measuring the platinum thermal resistance. Furthermore, the MAX31865’s third-order digital “sin c” filter provides the input noise rejection function, thus reducing the 50-Hz or 60-Hz power supply noise, including harmonics of the power supply’s fundamental frequency, by 82 dB. The physical map of the used temperature module is shown in Figure 2.

To measure the RTD resistance, the MAX31865 connects a reference resistor (RREF) in series with the RTD, and applies a bias voltage to the RREF. Then, the reference resistor’s current flows through the RTD. The voltage across the reference resistor represents the reference voltage for the ADC. Both ends of the RTD resistor are connected to the ADC differential inputs (PT+ and PT−), and thus, the digital output (Or) generated by the ADC is equal to the ratio of the RTD resistance to the reference resistance, which can be expressed by:

Or = RTD/RREF.(1)

For the reference resistor, it is appropriate to set the resistance value to be four times the RTD value at the zero Celsius temperature.

Accordingly, for the PT1000, a 4-kΩ reference resistor is used. Assume A is the temperature coefficient of platinum thermal resistance, then it holds that:

RTD = Or × RREF/2^15^, (2)

T = (RTD − 1000)/A.(3)

Since this module uses the PT1000, A has a value of 3.85055.

**Figure 3:**
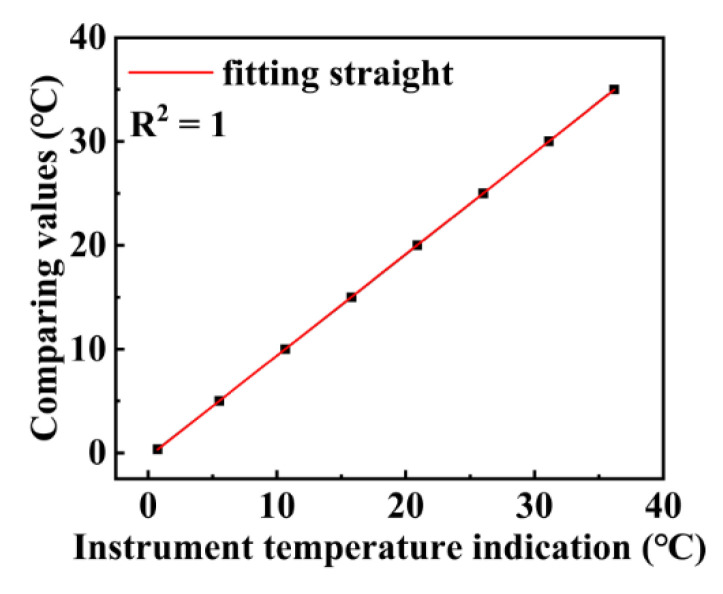
Results of the temperature sensor calibration experiment.

The module has been calibrated at the National Oceanographic Standards and Metrology Center, and the calibration results are provided in Figure 3.

Figure 3 shows the comparison between the temperature values measured by the instrument used in this work and that provided by the National Center of Ocean Standards and Metrology. As shown in Figure 3, there exists a highly-linear relationship between the two temperature values. Specifically, in the temperature range from −5 to 40 °C, the difference between the output temperature values of the two instruments is only 0.02 °C.

#### 2.2.2. Pressure Sensor Conditioning Circuit

The pressure conditioning module in this work uses a piezoresistive pressure sensor [12,13,14,15] with a measuring range of up to 3 MPa, to collect data on external pressure changes. For marine instruments, the measured pressure fundamentally represents the external water pressure, which depends on the water depth at which the instrument is installed. Particularly, a piezoresistive pressure sensor contains a sensitive element made of piezoresistive, which is arranged in a Wheatstone bridge configuration. When the sensitive element is not under pressure, the bridge remains balanced, and the Wheatstone bridge output is zero. In contrast, when the sensitive element is under pressure, the bridge goes out of balance and shows certain non-zero output. In addition, the pressure sensor can be excited either by a constant current source or a constant voltage source, while the bridge outputs a voltage signal proportional to the applied external pressure. In general, the piezoresistive pressure sensors have high sensitivity, precision, and allow miniaturization.

**Figure 4:**
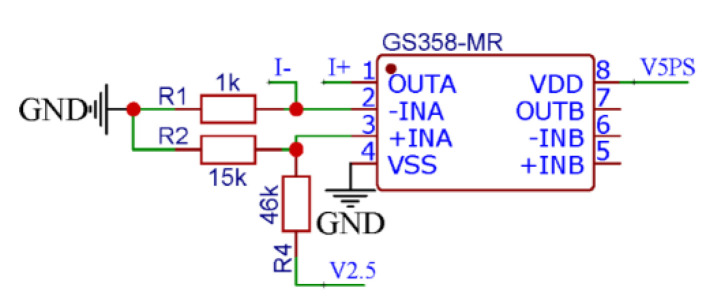
Schematic diagram of the constant current source circuit.

As shown in Figure 4, Gainsil’s GS358 is used to drive the pressure sensor with a 0.6 mA constant current. In particular, R2 and R4 are 15-kOhm and 46-kOhm resistors, respectively, and the reference voltage in the module is 2.5 V. Meanwhile, the positive input voltage of the operational amplifier is 0.6 V, and therefore, the negative input terminal voltage is also 0.6 V, due to the fundamental virtual short-circuit property of an operational amplifier. Since it is necessary to ensure the power supply of 0.6 mA constant current source to the sensor, R1 should have a resistance of 1-kOhm.

**Figure 5:**
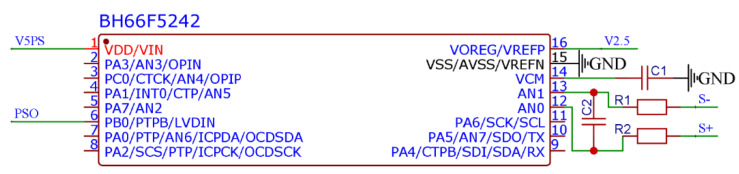
Schematic diagram of pulse width modulation circuit.

The pressure sensor has a differential output, where a single-channel voltage output is in a range of 70–130 mV; thus, the output amplitude of the differential signal of the voltage sensor is in a range of 0–60 mV. Due to the small amplitude of the voltage output, a high-precision ADC is essential. In addition, due to limited primary control resources of the storage circuit, this design performs the pulse width modulation on the voltage signal of the pressure sensor to output a PWM wave. As a result, the pressure changes are reflected in the duty cycle changes of a PWM wave. Therefore, a microcontroller must perform the signal conditioning on the pressure sensor. The BH66F5242 is an A/D type flash microcontroller with an eight-bit high-performance reduced instruction set and a built-in multi-channel 24-bit Delta Sigma type A/D converter. The BH66F5242 single-chip microcomputer is used in this work to adjust the pressure sensor signal, as shown in Figure 5, to meet the requirements of the ADC resolution and efficiently transform the voltage signal into a PWM signal.

Since the pressure sensor is designed based on the principle of the Wheatstone bridge, the output differential signal is relative to the related ground of the pressure sensor. Hence, the BH66F needs to use two ADCs to collect the differential signals, and then the differential processing is performed on the collected signals, to amplify the difference while reducing the influence of the common-mode noise. The amplified result is embedded in the function that changes the duty cycle of a PWM wave.

**Figure 6:**
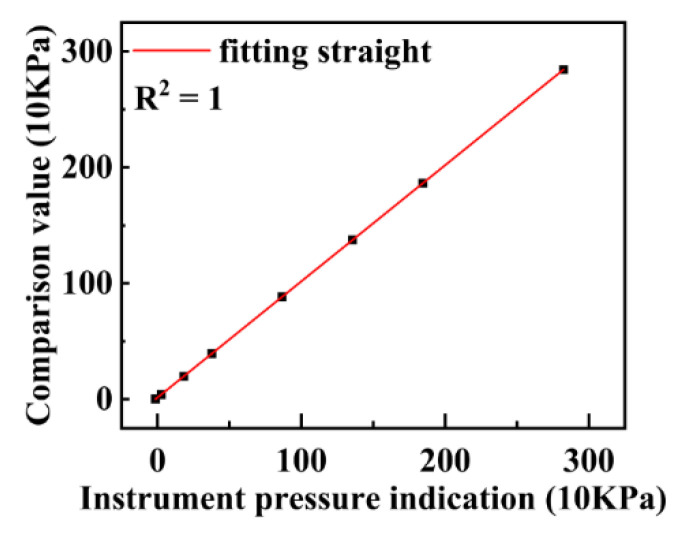
Results of the pressure sensor calibration experiment.

The water depth module was also calibrated in the National Center of Ocean Standards and Metrology. The vertical axis in Figure 6 represents the reference pressure value provided by the National Oceanographic Standard Metrology Center, whereas the horizontal axis is the instrument indication value output by the water depth module of the self-developed instrument. As shown in Figure 6, within a 3-MPa pressure range, the instrument indication value has a linear relationship with the reference value provided by the National Oceanographic Measurement Center. In the pressure range of 0–3 MPa, the instrument indication error is less than 2.4 kPa.

#### 2.2.3. Turbulence Sensor Conditioning Circuit

The turbulence measurement module used in this work collects the ocean microstructure turbulence signals at a scale of less than 1 m. Airfoil shear flow sensors [16,17,18,19] have been the most widely used in the field of ocean turbulence detection, and they use a piezoelectric transducer that converts the force signal of a turbulent flow into an electrical signal [20]. However, this type of sensor is a one-dimensional sensor, incapable of sensing multi-directional turbulent signals. In practical application scenarios, two sensors have been typically orthogonally placed to comprehensively observe the vectorial turbulence signal. However, this results in a lack of observational capabilities of instruments for the critical oceanic boundary layers and a lack of a sound theory behind the driving mechanisms of turbulence formation, mixing, and dissipation.

**Figure 7:**
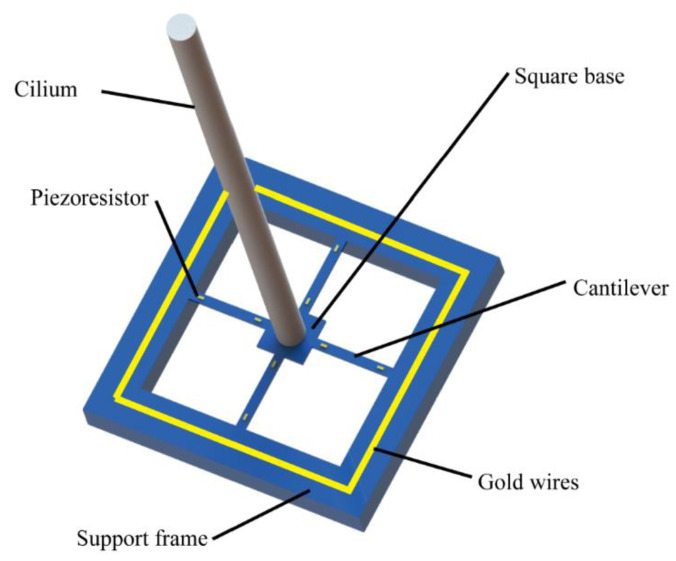
Structural design of a self-developed turbulence sensor.

The turbulence measurement module used in this work adopts a self-developed ciliary MEMS vector turbulence sensor [21], which is shown in Figure 7.

The proposed sensor consists of cilia, square cilia base, varistor, support frame, and on-chip gold wire. The cilia are located on the central base of the square shaped sensor structure and are connected to the support frame by means of cantilever beams. In addition, two varistors are placed on the upper side of each cantilever beam. Further, four cantilever beams are distributed in a cross shape, and cantilever beams in the same direction constitute a Wheatstone bridge [22]. Namely, the four cantilever beams are orthogonally placed and form two sets of Wheatstone bridges that sense the turbulent signals in mutually perpendicular directions. Moreover, the two Wheatstone bridges divide the measured turbulence signal acting on the cilia into *x* and *y* directions.

**Figure 8:**
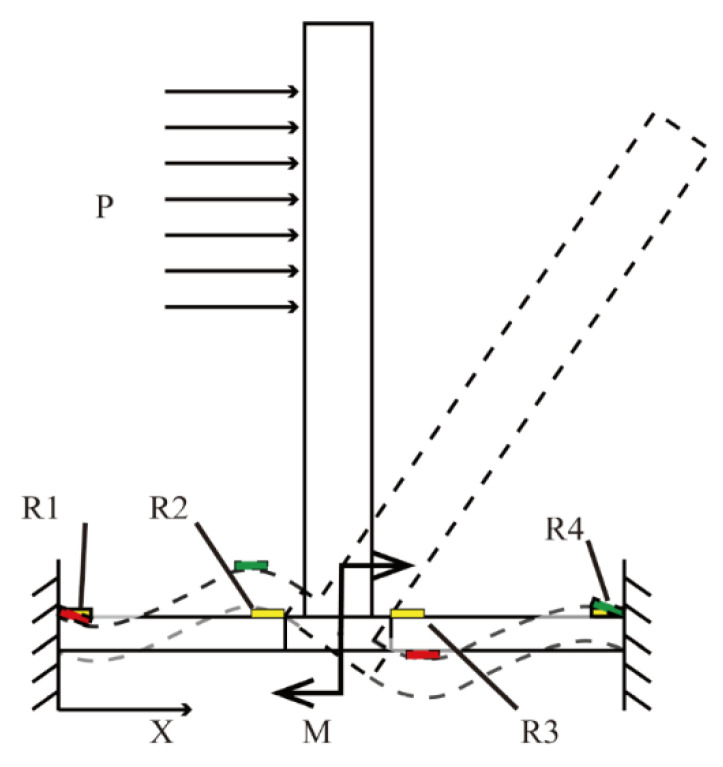
Schematic diagram of the turbulence impact in the positive *x*-axis direction of turbulence sensor.

As shown in Figure 8, when the cilia are subjected to the turbulent force from the negative *x*-axis direction, varistors R_1_ and R_3_ are subjected to the compressive force, while varistors R_2_ and R_4_ are subjected to the tensile force [23]. Similarly, when the cilia are subjected to a turbulent force in the positive *x*-axis direction, varistors R1 and R3 are subjected to the tensile force, and varistors R2 and R4 are subjected to the compressive force. This means that when the cilia are subjected to turbulent flow, the force component in the *x*-axis direction will cause the resistance values of varistors R1 and R3 to change in a similar way. Meanwhile, the resistances of varistors R2 and R4 also change in a similar way. However, the changing trends of R1 and R3 and those of R2 and R4 are opposite. By analogy, the resistances of varistors on the *y*-axis also show the same changing principle. On the Wheatstone bridge, two resistors with the same resistance-changing trend are placed on the opposite bridge arms, while the two resistors with opposite resistance-changing trends are placed on the adjacent bridge arms. The magnitudes of the turbulence vector in the two directions are measured by the voltage outputs of two orthogonal Wheatstone bridges. When the cilia are not affected by turbulence, the bridge remains balanced, since the varistors’ resistances are the same. However, when the cilia are subjected to turbulence, the varistors’ resistances change by ΔR, and the resistance change induces a proportional output voltage. The bridge output can be expressed as follows:

(4) $$V_{o-x}=\frac{\left(R_{1}+{\;\Delta R}_{1}\right)\left(R_{3\;}+{\;\Delta R}_{3}\right)-\left(R_{2}-{\;\Delta R}_{2}\right)\left(R_{4}-{\;\Delta R}_{4}\right)}{\left(R_{1}+{\;\Delta R}_{1}+{\;R}_{2}-{\;\Delta R}_{2}\right)\left(R_{3}+{\;\Delta R}_{3\;}+{\;R}_{4}-{\;\Delta R}_{4}\right)}V_{\mathrm{in}}$$

The Wheatstone bridge can effectively suppress the common mode noise, amplify differential mode signal, and improve sensor’ sensitivity.

Turbulence sensors have been commonly used in underwater environments, and the sensor’s output signal can be easily disturbed by a working environment. Therefore, the sensor output signal cannot be directly collected; it must be first amplified and filtered.

**Figure 9:**
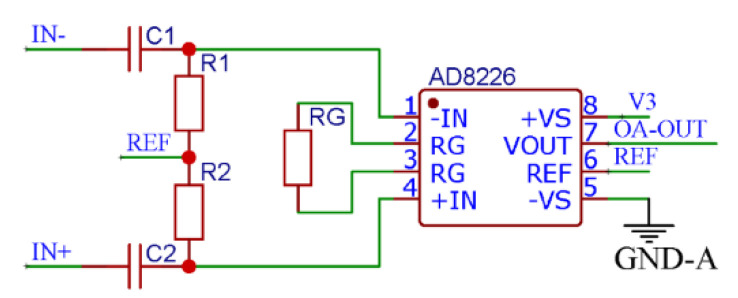
Circuit diagram of differential amplification of the turbulence sensor signal.

For the two Wheatstone bridges of the self-developed sensor, the sensor has two pairs of differential signal outputs; thus, it is necessary to perform differential processing on the output signals of each of the Wheatstone bridges. Since an ADC can collect only positive voltage signals in the range of 0–3 V, when performing the ADC, the module first pulls up the collected signal by 1.5-V. To provide a 1.5-V offset, a LP2985AIM5-1.5 chip, Texas Instruments, with a wide input voltage range, large precision voltage output, and strong peak current capability is used. In addition, an AD8226 instrumentation amplifier, Analog Devices, features low offset and high common-mode rejection, making it an excellent choice for amplifying the bridge circuit output. The amplification gain of the AD8226 is RG = 49.4 kΩ/(G − 1), and it achieves an optimal signal-to-noise ratio when the AD8226 gain resistor’s resistance is set to 100 Ohm, where it amplifies the signal 495 times. As shown in Figure 9, the pull-up signal should be differential and amplified with a 1.5-V reference signal. Therefore, 1.5-V is input to the reference terminal of AD8226.

**Figure 10:**
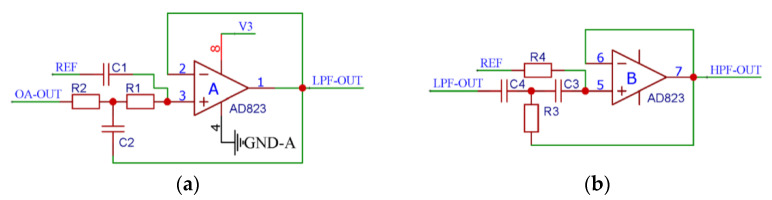
Schematic diagrams of the Butterworth filter circuits: (**a**) high-pass filter; (**b**) low-pass filter.

Two ion implantations are performed when fabricating the varistor and ohmic contact regions on the cantilever beam of the self-developed turbulence sensor. However, due to certain process defects, such as uneven mixing, the initial resistance value of the varistor could be inconsistent and the resistance value of the ohmic contact area could be too high. As a result, the turbulence sensor can still show a direct current (DC) signal output even when it is not excited by external turbulence. Therefore, the sensor signal needs to be high-pass filtered, as shown in Figure 10a. The turbulence sensors work in marine environments that are affected by environmental noise, such as hydrodynamic and biological noise. The micro-scale turbulent movement concentrates around 500 Hz, generally below 1 kHz. Thus, it is also necessary to perform low-pass filtering on the sensor signal, as shown in Figure 10b.

The AD823 is a filter chip with the characteristics of low distortion and low noise, and it has been adopted in the proposed system. To obtain smooth filtered signals, this study adopts the Butterworth filter that has a flat frequency response curve in the passband.

The cut-off frequency of the filter is given by:

(5) $$f_{c}=\frac{1}{2\pi \sqrt{R_{1}R_{2}C_{1}C_{2}}}$$

The turbulence sensor signal is amplified and filtered, and the corresponding output signal is in the range of 0–3 V. According to the Nyquist sampling theorem, the ADC sampling frequency is set to 4 kHz.

**Figure 11:**
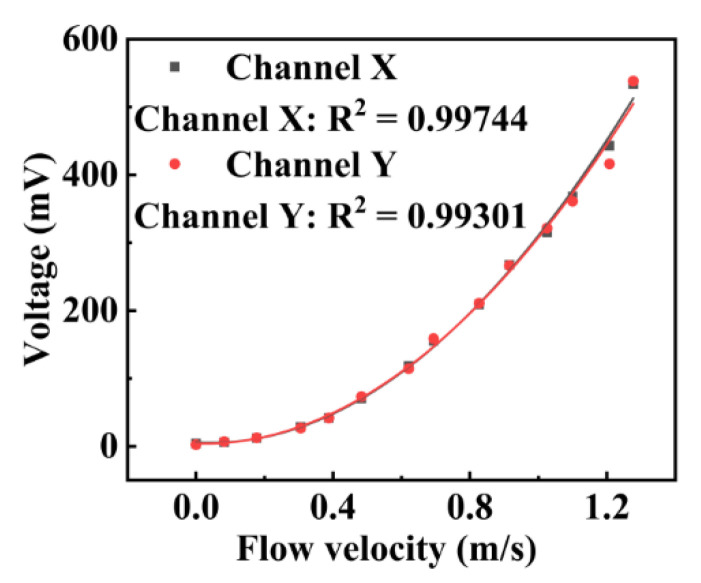
Results of the turbulence calibration experiment.

Next, the self-developed turbulence sensor has been calibrated, as shown in Figure 11, where the vertical axis represents the indication value of the turbulence sensor module of the self-developed instrument, and the horizontal axis denotes the turbulent jet velocity recorded by the turbulence sensor calibration system. In Figure 11, black points represent the turbulence sensor’s *x*-axis calibration data, while red points denote the *y*-axis calibration data. As shown in Figure 11, the relationship between the sensor output signal and the turbulent flow velocity follows the quadratic polynomial law. Furthermore, the *x*- and *y*-axis calibration data coincide, indicating that the two sensor outputs are highly consistent.

#### 2.2.4. Conductivity Sensor Conditioning Circuit

The conductivity module of the proposed system measures the conductivity of liquids in milli-siemens per centimeter (mS/cm). Through the liquid conductivity measurement, combined with the temperature and pressure measurement data, the salinity of the liquid can be obtained. Conductivity sensors determine the conductivity of liquids by measuring the voltage and current between their sensing electrodes. For instance, a two-electrode conductivity sensor calculates the liquid conductivity by applying a specific constant voltage between its two electrodes and measuring the corresponding electrode current. However, this measurement method suffers from errors caused by electrode polarization [24,25,26]. Therefore, the proposed module adopts a four-electrode conductivity sensor [27] to separate the current and voltage electrodes. Namely, the voltage electrodes are between the current electrodes, and the four electrodes are in the same plane. Notably, the distance between the inner electrodes should be maximized to ensure that the voltage measured by the voltage electrodes is significant when the current electrode is excited, which simplifies the subsequent calculation. However, since the inner electrodes have a large input impedance, they will not produce any polarization when measuring the voltage, and thus, the current measured by the outer electrode will not be affected. In addition, when a four-electrode conductivity sensor is used, since the voltage measuring electrode is separated from the current measuring electrode, the influence of the electrode polarization on a conductivity sensor can be avoided.

**Figure 12:**
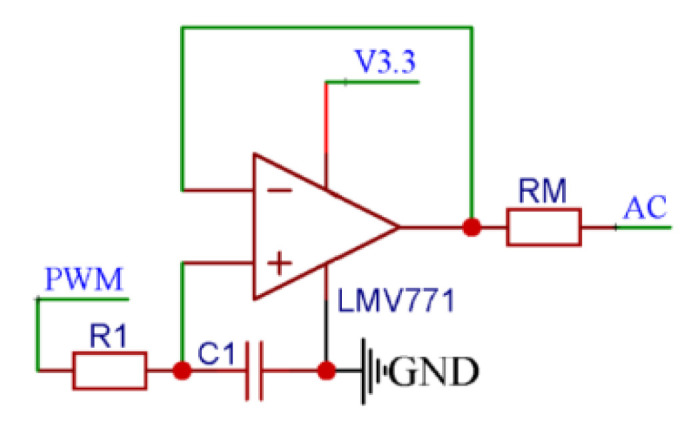
The AC excitation signal generation circuit.

During the conductivity measurements process, the DC excitation and low-frequency alternating current (AC) excitation can lead to significant errors, and related studies have shown that when the excitation signal frequency is higher than 1 kHz, the conductivity measurement results tend to be stable with high reliability. The conductivity signal conditioning chip used in this design is an STM32F0 with a DAC of 12 bits, but its resolution does not meet the requirements for efficient MCU reconstruction of the excitation signal. Therefore, the proposed module uses a high-frequency PWM wave to output the AC excitation signal through the capacitor oscillator. As shown in Figure 12, the PWM wave output by the STM32F0 oscillated between R1 and C1, which yields a triangular wave input signal at the positive input end of the voltage follower. Then, the input signal is shaped by the voltage follower and output to create a triangular wave excitation signal with high-drive capability.

To control the voltage signal measured by the inner electrode and the current signal measured by the current electrode in their measurable ranges, the liquid to be tested is matched with a specific RM resistance value. This guarantees that when the conductivity is high, the current signal will not be distorted at the peaks, and when the conductivity is low, the voltage signal will not be distorted at the peaks.

**Figure 13:**
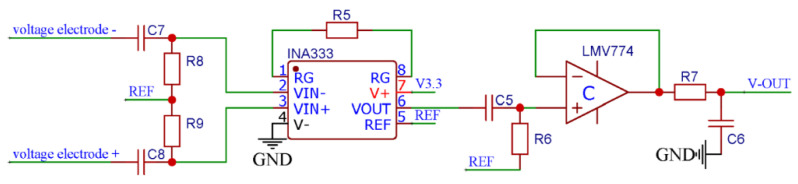
The block scheme of a differential amplifier circuit for the inner electrode signal.

The conductivity module uses a differential amplifier circuit to convert two-channel weak internal electrode voltage signals into a single-channel voltage output signal, as shown in Figure 13. A INA333 instrumentation amplifier from Texas Instruments, with the characteristics of low power consumption and high precision, is used in this work, and its gain is obtained by: G = 1 + (100k/R5). This amplifier differentially amplifies the AC voltage signal measured by the inner electrode. By adjusting the resistance value of gain resistor R5, the differential signal is amplified by 26 times, reflecting the voltage difference between the internal electrodes.

**Figure 14:**
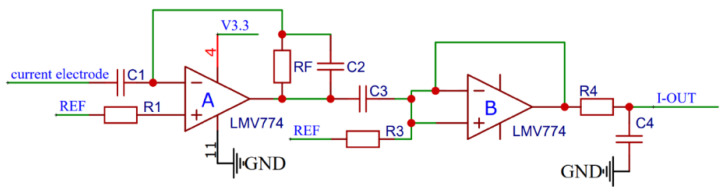
Schematic diagram of a current signal conditioning circuit.

In addition, the current signal is converted into a voltage signal using a capacitor to measure the liquid current, as presented in Figure 14. At this time, the potential at the negative input terminal (pin 2) of operational amplifier A is consistent with the loop current change. Assume that the liquid to be tested is an unknown resistance RK, which is connected in series with the matching resistor RM. Moreover, assume that a negative feedback amplifier resistor RF is used to amplify the potential of pin 2. The RF resistance value should be set in a way that the current signal after conversion and amplification is not distorted.

Since the excitation signal of the conductivity sensor is an AC signal, the signals measured at the voltage and current electrodes are also AC signals. Moreover, the excitation signal has a frequency of 1 kHz. To reproduce the periodic signal change, according to the Nyquist sampling theorem, the sampling frequency of the ADC should not be lower than twice the excitation frequency. In terms of the excitation frequency, the higher the sampling frequency is, the better the signal to be tested can be reproduced. Thus, the module uses an 8 kHz sampling frequency to collect the signal to be measured. The discrete signals collected by the module include signal amplitude, signal frequency, and DC component of the bias voltage. It should be noted that when dealing with discrete AC signals, the DC component of the voltage signal is useless. Therefore, the first step in the single-chip processing of the collected signal is to remove the DC component from the collected signal. Since the frequency of the excitation signal is constant and the frequency of the signal to be measured is related only to the excitation frequency, the module only needs to integrate the signal amplitude. Due to the particularity of an AC signal, the absolute value of the AC signal after removing the DC component is integrated. To characterize the signal size better, the acquisition duration needs to cover at least one excitation period. In addition, the proposed conductivity module averages the integrated results at every 4000 sets of collected data, to reduce the measurement error.

After the conductivity module differentiates, amplifies, and integrates the voltage electrode signal, data S_u_ are obtained. Similarly, after the current signal is amplified and integrated, data S_i_ are obtained. Then, the conductivity can be expressed as follows:

σ = k × S_i_/S_u_, (6)

σ is the liquid conductivity, and k is the electrode constant.

**Figure 15:**
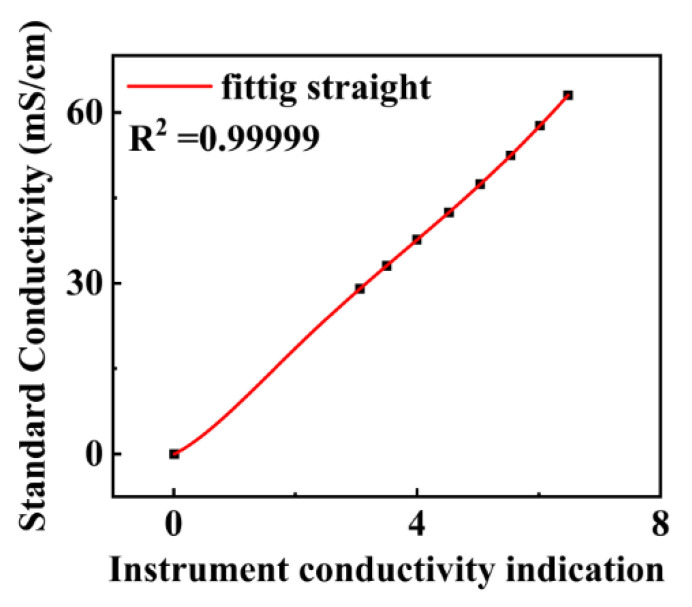
Results of the conductivity calibration experiment.

The conductivity module was calibrated at the National Center of Ocean Standards and Metrology. In Figure 15, the *x*-axis denotes the electrical conductivity output data of the self-developed instrument, and the *y*-axis shows the reference electrical conductivity value given by the National Marine Standard Metrology Center. In the range of 0–70 mS/cm, the self-developed instrument shows an error of less than 0.073 mS/cm.

#### 2.2.5. Self-Capacitance Storage Circuit

The proposed system uses four different sensors: a pressure sensor, a temperature sensor, a conductivity sensor, and a turbulence sensor. According to the actual needs, the proposed system has different requirements for the operating frequencies of four detectors. For the pressure, temperature, and conductivity sensors, the storage frequency is set to 1 Hz, and for the turbulence sensor, the storage frequency is set to 4 kHz.

Since the storage rate of the turbulence sensor is 4 kHz, the storage unit needs to have the characteristics of a large storage capacity and a high data transmission rate. A trans-flash (TF) card offers the advantages of large storage capacity, small size, and high transmission rate, and thus, the proposed system adopts the TF card as a storage unit.

To increase data readability, the proposed system uses a FAT file management system. The FAT file management system is independent of the hardware platform, easy to transplant, and has independent buffers.

Obviously, the data storage rate of the turbulence sensor is much higher than that of the other three sensors, so the turbulence sensor’s data are stored separately. By using the FAT file management system, the proposed system independently frames the turbulence sensor data and saves the file name in the format of the current hour value in an independent folder. Moreover, as the storage rates of the other three sensors are the same, the data of these three sensors are uniformly framed and stored in the same folder having the current hour value as a file name.

### 2.3. Performance

**Figure 16:**
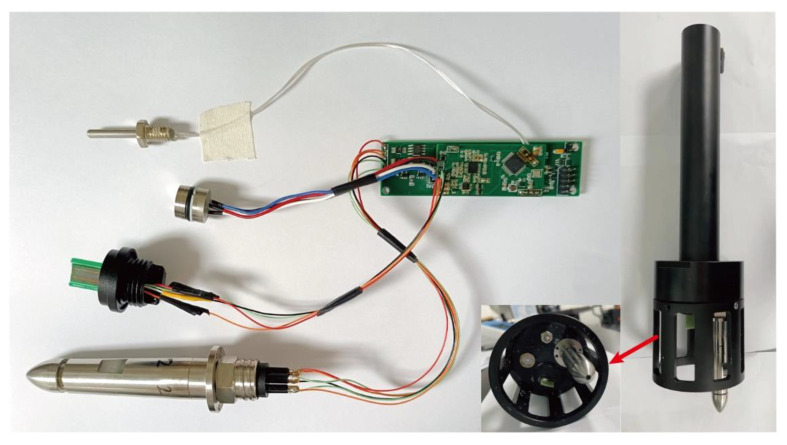
A physical diagram of the designed circuit.

The overall designed circuit is shown in Figure 16.

The entire hardware system was packaged into a device, as shown in Figure 16. The temperature, pressure, and conductivity probes were installed on the same plane, and the turbulence sensor probe was mounted on a plane that was higher than the other plane to ensure that the collected turbulence data would not be affected by the presence of the other probes.

**Figure 17:**
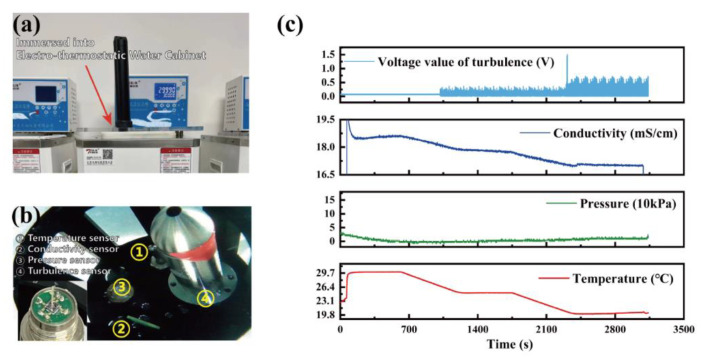
Four-parameter space-time synchronization test: (**a**) experimental apparatus; (**b**) sensors in water cabinet; (**c**) experimental results.

As shown in Figure 17a, the entire device was placed in an electro-thermostatic water cabinet with a water circulation function for comprehensive testing. The photo of the device in the electro-thermostatic water cabinet is presented in Figure 17b. The KCl solution was placed in the water cabinet. There were three constant temperature points during the experiment: 30 °C, 25 °C, and 20 °C. Each temperature point was kept constant for approximately five minutes. When the temperature was higher than 26 °C, the water circulation function of the water cabinet was turned off; when the temperature was lower than 26 °C, the water circulation function was turned on. At a temperature of 20 °C, the water cycle was turned on, so a stirring rod was used to rapidly stir the water in the constant-temperature tank to increase the water flow rate.

The experimental results are shown in Figure 17c.

The conductivity data were trended in line with the temperature data. When the device was put into the water, the temperature suddenly changed from room to water temperature. At the same time, the conductivity sensor entered the KCl solution from the air, and the conductivity data suddenly changed. When the water temperature was maintained, the conductivity data were also maintained. Similarly, when the water temperature decreased, the conductivity data also decreased. The spatiotemporal consistency of the conductivity and temperature sensor was demonstrated.

In the experiment process, when the temperature was higher than 26 °C, the turbulence sensor was in a non-turbulent environment, and the overall output should be zero. However, since the piezoresistors of the sensors could not be kept entirely consistent, the collected turbulence sensor data were higher than zero, approximately 0.07 V. When the temperature was less than 26 °C, the output of the turbulence sensor significantly increased with the water flow rate. At a temperature of 20 °C, the water flow rate was higher than that of the previous stage, and the output of the turbulence sensor continued to increase. This demonstrated the spatiotemporal consistency of the temperature and turbulence sensors.

The pressure sensor could not be effectively pressurized due to the shallowness of the water cabinet; therefore, in the experiment, the pressure output signal remained unchanged. In addition, this experiment could not reflect the spatio-temporal consistency between the pressure sensor and other sensors, so the pressure calibration experiment was used to verify the relation between the pressure sensor and other sensors.

**Figure 18:**
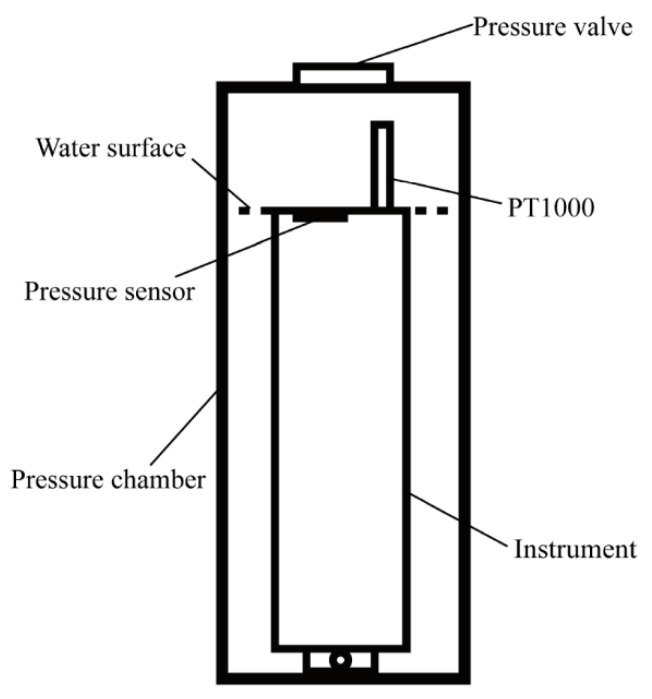
Schematic diagram of the instrument placement in the pressure calibration experiment.

The pressure calibration experiment was conducted at the National Center of Ocean Standards and Metrology. Before starting the investigation, the combined temperature and depth sensor (TD) was placed in a room at a constant temperature for three hours to ensure a constant temperature of the instrument at the beginning of the experiment. Afterwards, the device was powered on and placed in the pressure chamber, as shown in Figure 18.

Specifically, the instrument was placed in a pressure-resistant chamber, and the pressure sensor was leveled with the water surface. Further, the PT1000 was placed on the water surface and exposed to the gas in the pressure-resistant chamber. Furthermore, the calibration of the eight pressure points of the experimental instrument was completed by performing the stepwise pressurization and stepwise decompression of the pressure chamber seven times.

**Figure 19:**
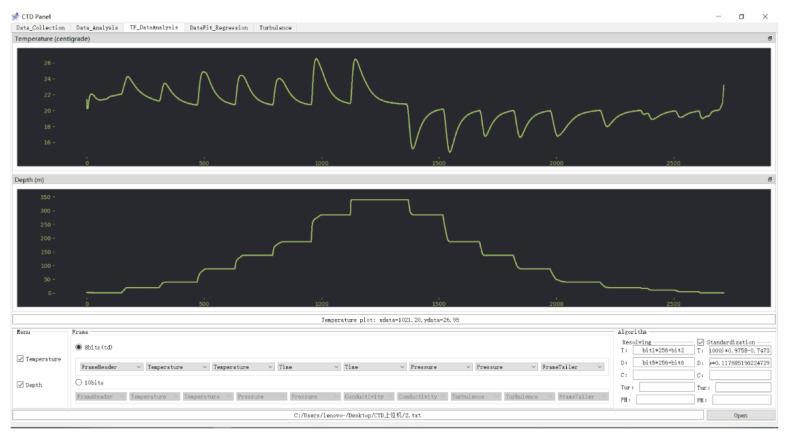
Interface diagram of the host computer.

**Figure 20:**
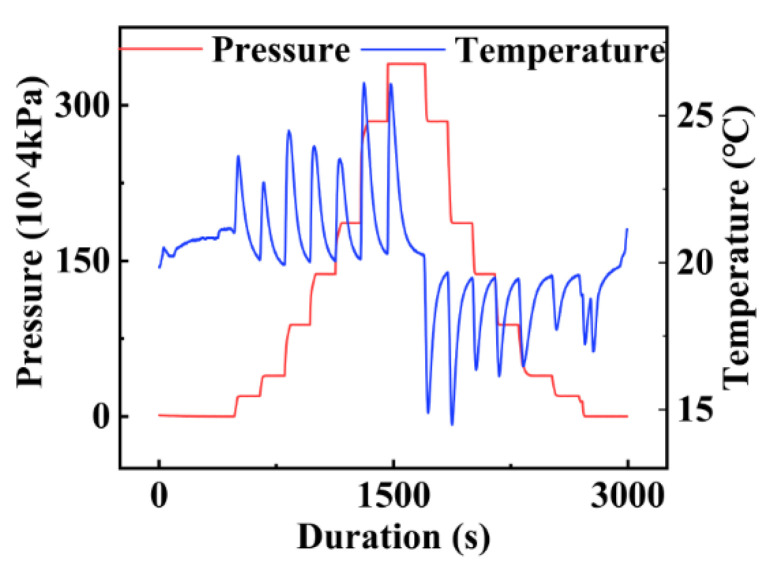
Comparison of the temperature and pressure results of the pressure calibration experiment.

A host computer was used to process the data stored in the TF card, and its interface is presented in Figure 19. First, the system completed synchronous data recording from different sensors. Next, the data processed by the host computer were redrawn, as shown in Figure 20. Each pressurization step corresponded to the compression of the gas in the cabin, which caused the cabin temperature to sharply increase. Due to the constant temperature of the experimental environment and the large specific heat capacity of the water, the temperature dropped to the ambient temperature after the pressurization. In contrast, during the decompression process, due to the pressure reduction, the gas expanded, and the temperature in the cabin sharply dropped. Following the decompression, the cabin temperature increased to match the ambient temperature. In the pressure calibration experiment, the pressure and release processes were performed seven times. During the pressurization process, the pressure increased seven times, and the rising edge in each case was consistent with the rising edge of the temperature change. Meanwhile, during the pressure release process, the pressure was released seven times. However, due to mechanical problems, the theoretical last pressure release process actually corresponds to two pressure release steps, and thus, the temperature fluctuated eight times in total during the entire pressure release process. The fluctuations were highly consistent with the pressure changes. The pressure calibration experiment reflected the synchronization of the measurement system.

The results of the two abovementioned experiments verified that the conductivity, turbulence, pressure, and temperature sensors were consistent in both time and space domains.

### 2.4. Perspectives

**Figure 21:**
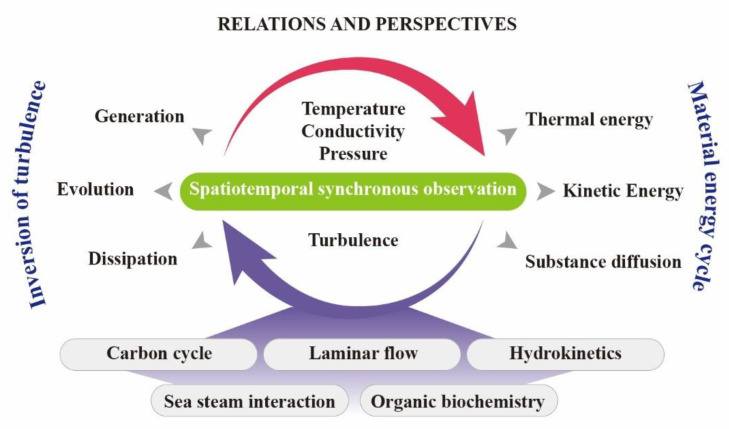
The correlation and perspectives of the spatiotemporal synchronous observation data of temperature, conductivity, pressure and turbulence data.

As depicted in Figure 21, the spatiotemporal synchronous observation of temperature, conductivity, pressure and turbulence is beneficial to study the mutual-driven mechanism of these four basic dynamic parameters. For instance, temperature and salinity differences in water can create turbulence, whose rate varies at different depths. The generated turbulence, in turn, accelerates the rebalance. The generation, evolution, and dissipation of turbulence all stem from the interaction between temperature and salinity differences in the water. It can also be used for turbulence inversion simulation, and turbulent mixing study on the meter or ten-meter scale after multiple collection points have been arranged in space to form a three-dimensional array. Furthermore, turbulence and these differences can strengthen the material energy cycle, which refers to thermal and kinetic energy transfer and matter diffusion. Therefore, the spatiotemporal synchronous studies of the interaction mechanism of dynamic parameters of seawater could promote marine research, on the carbon cycle, laminar flow, hydrokinetics, sea steam interaction, and organic biochemistry.

## 3. Conclusions

This paper presents a signal processing and data storage circuit that includes temperature, pressure, conductivity, and turbulence sensors. By using the proposed system to obtain data on a TF card and drawing the corresponding graph, the depth profile’s temperature, conductivity, and turbulence data can be intuitively analyzed. In addition, the proposed system uses the laboratory-made four-electrode conductivity sensor and ciliated turbulence sensor to achieve an integrated package with the multi-sensor probes and conditioning memory circuits. It should be noted that a third party has standardized and calibrated the temperature, salinity and depth sensors. As a result, all three of the four sensors can meet China’s national second-class standard for the CTD. In addition, the feasibility of the self-developed turbulence sensor was verified by laboratory calibration experiments. The experimental results show that the proposed system can simultaneously perform the space-time acquisition and storage of temperature, depth, conductivity, and turbulence parameters. Therefore, the target system benefits marine research, such as marine physics, marine biology, and marine meteorology.

## Data Availability

The data presented in this study are available on request from the corresponding author.
